# Awake tracheal intubation: Videolaryngoscopy in a pediatric institution: Use of guidelines and multidisciplinary team preparation to facilitate performance of an unfamiliar technique in a pediatric setting

**DOI:** 10.1002/ccr3.5466

**Published:** 2022-02-18

**Authors:** James Richard Skelly, Jessica Wauchope, Michael Collreavey, Bill Walsh

**Affiliations:** ^1^ Department of Anaesthesiology and Pain Medicine Children’s Health Ireland at Temple St. Dublin 1 Ireland; ^2^ Department of Otolaryngology Children’s Health Ireland at Temple St. Dublin 1 Ireland; ^3^ 8797 School of Medicine University College Dublin Dublin 1 Ireland

**Keywords:** airway management, guidelines, intubation, pediatrics

## Abstract

Formal guidelines for awake tracheal intubation have recently been published providing a streamlined process for the first time. We present a case of awake videolaryngoscopy in the pediatric setting, not previously reported. Application of guidelines and careful team preparation facilitated performance of a novel technique in our pediatric institution. A multidisciplinary approach with ENT colleagues provided a patient‐specific airway management plan for a rare airway pathology.

## Graphical abstract

Awake tracheal intubation is the gold standard for the anticipated difficult airway. The process has recently been formalized with the publication of the first guidelines providing a standardized technique. Application of guidelines and careful team preparation can facilitate performance of awake intubation in centers where it is uncommonly encountered such as a pediatric institution.

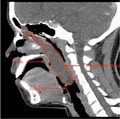

## 1 CASE REPORT

**FIGURE 1:**
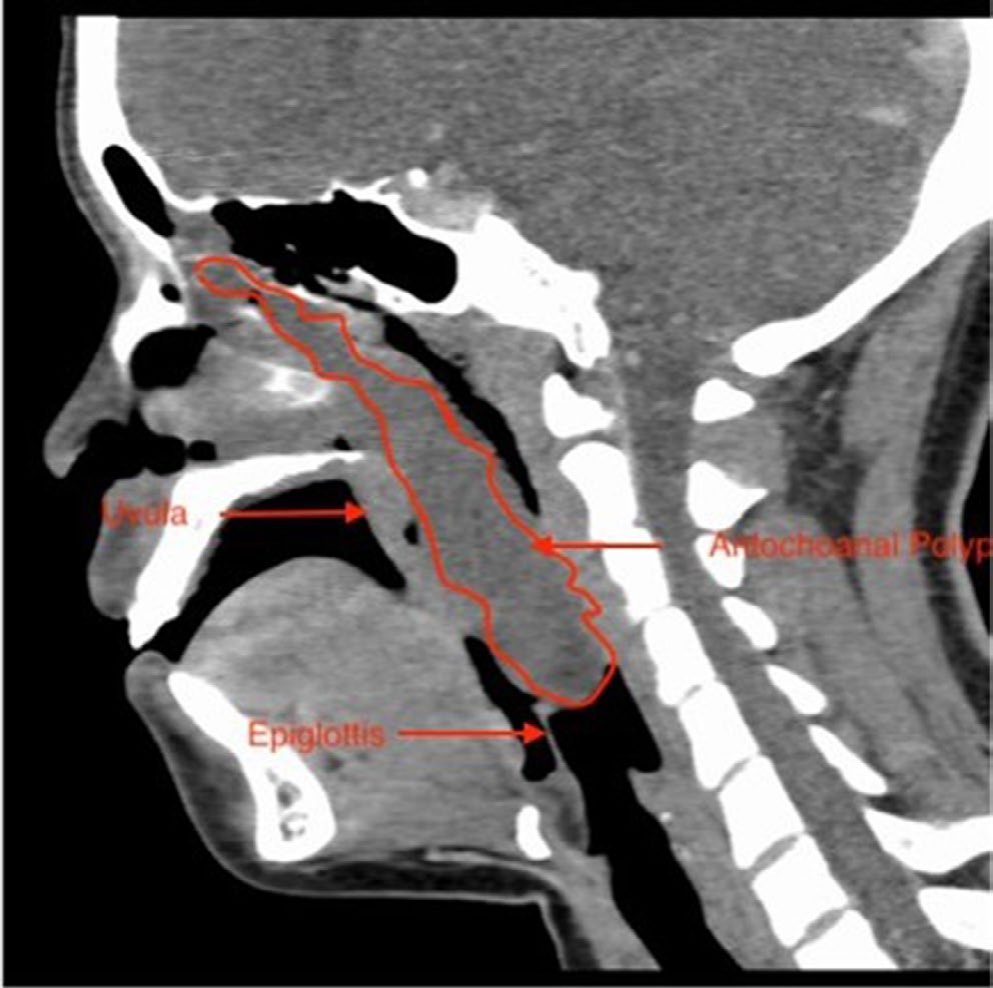
Sagittal CT image showing the extent of airway obstruction caused by the polyp

A 45 kg 16‐year‐old woman was admitted urgently for resection of an obstructing nasopharyngeal tumor, a suspected antrochoanal polyp. The mass extended from the right maxillary sinus through the nasal cavity into the oropharynx where it impinged upon the epiglottis (Figure 1). The soft palate was deviated antero‐inferiorly, partially occluding the airway. It measured 2.9 × 3.9 cm in the oropharynx and 12.4 cm in length.

The patient described altered phonation as well as dyspnea in the supine position. This was alleviated by lying on her side. Flexible nasendoscopy confirmed an extensive mass occupying both the naso‐ and oropharynx extending to the epiglottis. Clinical examination of the airway revealed a Mallampati score of I, good mouth opening, unrestricted neck movement and a thyromental distance of greater than 6.5 cm. The mass was visible protruding posterior to the uvula.

Intravenous access was obtained, and standard monitoring was applied. The ear, nose, and throat (ENT) surgical team was in the room, and a tracheostomy set was open. Sedation was initiated with a remifentanil infusion at 0.05 mcg/kg/min. The vocal cords were anesthetized with 4 ml of nebulized 4% lidocaine (West‐Ward Pharmaceuticals Corp., USA) (40% of maximum recommended dose). Nasal high flow oxygen (30 L/min) was then commenced and the oropharynx was anesthetized with co‐phenylcaine (ENT Technologies Pty Ltd, Australia) spray over a 5 min period (10 sprays, 50 mg lidocaine, 12% of maximum recommended dose). The videolaryngoscope (C‐MAC, Storz 8403ZX, Tuttlingen, Germany) was gently inserted and advanced as tolerated. Verbal communication was maintained throughout. Upon visualization of the larynx, the glottis was further anesthetized with 2 ml of 4% lidocaine (20% of maximum recommended dose) using a mucosal atomization device (MADgic laryngo‐tracheal mucosal atomization device, Teleflex Medical, USA). A spray above the glottis elicited a strong cough reflex. Once this subsided the atomization device was advanced through the cords and the remaining anesthetic was applied to the subglottis (1ml of 4% lidocaine, 6% of maximum recommended dose). A size 6 endotracheal tube (ETT) mounted on an intubating stylet was then passed on first attempt. The patient was comfortable with the ETT in situ. Once end‐tidal CO_2_ was attained anesthesia was induced with a target controlled infusion (TCI) of propofol.

Awake tracheal intubation:video laryngoscopy (ATI:VL) has not previously been described in the pediatric literature. Recent guidelines^1^ have streamlined a suggested awake tracheal intubation (ATI) procedure for the first time. They have also formalized ATI:VL as a viable airway management strategy. The guidelines help to promote the technique and reduce anxiety among practitioners in settings where it is uncommonly encountered such as our tertiary pediatric hospital.

Antrochoanal polyps are benign lesions arising from the maxillary sinus.^2^ They are the cause of 33% of nasal polyps in children.^3^ Large polyps have previously been reported to cause obstructive sleep apnea in children^4^, ^5^ but challenging airway management has not been described.

Potentially impossible bag mask ventilation necessitated a cautious approach to airway management in our patient. The mobile polyp was abutting the larynx. Hanging from its stalk in the nasopharynx it had the potential to act as a ball and cage valve with the application of proximal positive pressure. Spontaneous ventilation was deemed to be essential to the safe management of this child's airway.

A four step airway management plan was devised with the ENT team. Plan A was ATI:VL, not previously performed in our pediatric institution. Indeed, some of our nursing colleagues were completely unfamiliar with awake intubation. An in‐depth team brief was undertaken with consideration of the plan, its rationale, the step‐by‐step process, and each team member's role. The rigidity of the laryngoscope blade was postulated to provide a means to deflect the mobile mass from the path of the tube. A flexible bronchoscope may have failed to progress beyond the obstructing polyp. Plan A was dependent on the co‐operation of the child. Plans B to D involved maintenance of spontaneous ventilation with total intravenous anesthesia (TIVA), should plan A have failed. Plans B to D were as follows: videolaryngoscopy, rigid bronchoscopy, and front of neck access, respectively. A tracheostomy set had been prepared.

The recent ATI guidelines describe 9 mg/kg of lidocaine as an absolute upper limit. We set a limit of 7 mg/kg and found this adequate to provide the requisite airway anesthesia. Indeed, it allowed for a remarkable tolerance of the endotracheal tube.

We feel this technique could be utilized in any scenario in which a difficult intubation is predicted in co‐operative older children, in particular in congenital conditions including the mucopolysaccharidoses and Goldenhaar syndrome. The technique would also lend itself to situations where positive pressure ventilation could lead to complete loss of airway such as tracheal foreign body or a mediastinal mass.

In conclusion, we present a case of ATI:VL, which we believe to be the first in the pediatric setting. Recently published ATI guidelines helped allay anxiety in performing this technique in a center where it has not been encountered. An MDT approach provided a safe stepwise contingency plan should this technique have failed.

## 2 LEARNING POINTS

1. The Awake tracheal intubation process has recently been formalized with the publication of the first guidelines providing a standardized technique.
2. Application of guidelines and careful team preparation facilitate performance of awake intubation in a center where it is uncommonly encountered such as a pediatric institution.
3. An MDT approach with ENT colleagues is useful in formalizing a patient‐specific airway management plan when encountering rare airway pathology.

## CONFLICTS OF INTEREST

The authors have no competing interests, financial, or otherwise.

## AUTHOR CONTRIBUTIONS

JR Skelly main author and researcher. J Wauchope and M Collreavey contributing researcher and author. B Walsh main author and project coordinator.

## CONSENT

Written informed consent was obtained from the patient to publish this report in accordance with the journal's patient consent policy.
